# Editorial: Towards plant-based foods: from plant biodiversity to discovery of sensorially active compounds

**DOI:** 10.3389/fpls.2023.1240745

**Published:** 2023-07-31

**Authors:** Gholamreza Khaksar, Takayuki Tohge, Supaart Sirikantaramas

**Affiliations:** ^1^ Center of Excellence in Molecular Crop, Department of Biochemistry, Faculty of Science, Chulalongkorn University, Bangkok, Thailand; ^2^ Graduate School of Biological Science, Nara Institute of Science and Technology (NAIST), Ikoma, Japan; ^3^ Omics Sciences and Bioinformatics Center, Chulalongkorn University, Bangkok, Thailand

**Keywords:** chemodiversity, flavor-related compounds, metabolism, metabolite profiling, plant-based food (PBF)

## Introduction

An ever-growing interest among health-conscious consumers has resulted in greater attention to healthy diets of natural origin (Aleksandrowicz et al., 2016). Previous studies have well documented a strong link between an increased intake of plant-based foods (PBFs) and lower risk factors of major chronic diseases in humans. Various health-beneficial properties inherent to PBFs have been attributed to their rich content of bioactive compounds. Texture, flavor, and color are major sensory characteristics of a food product, among which flavor (taste and aroma) is considered the key factor with the greatest effect on consumer appeal and acceptance. The flavor profiles of PBFs are mainly controlled by their biochemical composition, which also impacts other important properties of PBFs, such as shelf life and nutritional and economic value (Goff and Klee, 2006; Pavagadhi and Swarup, 2020).

Plants harbor a rich metabolic diversity, with more than 200,000 metabolites in various plant tissues, such as fruit, seed, leaf, and stem (Wurtzel and Kutchan, 2016). Some of these metabolites along with other bioactive compounds, such as peptides, contribute to the flavors of PBFs, creating numerous unique flavors, e.g., the contribution of glutathione to umami and glutamate to kokumi taste. Of note, breeding programs have mainly tried to enhance yield, resulting in significant deterioration in the flavor of many PBFs, which is of major concern among breeders and food scientists (Pavagadhi and Swarup, 2020).

Nowadays, analytical approaches are mainly based on physicochemical estimations and sensory-dependent tests to investigate flavor-associated characteristics in PBFs. However, they do not provide enough understanding of the biochemical composition or metabolic aspects related to unique flavor profiles. There exists a need for updated means to give us more quantitative information on the biochemical composition of PBFs as consumers’ expectations grow continually regarding food quality. Accordingly, metabolomics can be utilized as a promising approach to provide a systematic understanding of flavor-associated metabolites. Therefore, the scope of metabolomics in this area should not only focus on the discovery of flavor-associated metabolites but also linking those metabolites to taste and aroma to be further used for developing new PBFs with enhanced flavor and biomarker development related to unique flavors. Notably, integrating flavor-associated metabolic profiling with other up-to-date means derived from big data looks promising for this research area (Zhu et al., 2019).

This Research Topic presents five research articles contributed by 41 authors. Here, we summarize these papers.

## Identification of flavor-related compounds in economic crops

In this study (Peng et al.), mango fruit pulps at the mature-green (MG) and tree-ripe (TR) stages were metabolically profiled, which led to the identification of 309 different metabolites, such as organic acids, amino acids, and phenolic compounds, along with 84 volatile organic compounds (VOCs). Moreover, carotenoid contents were also screened, revealing 68 carotenoids, among which major levels of zeaxanthin palmitate and (E/Z)-phytoene were reported for the first time in mango fruit (*Mangifera indica*). The findings of this study revealed key metabolic shifts during the fruit development from the MG to TR stage of fruit ripening, which may contribute to changes in fruit flavor.

In Thailand, there are numerous colored rice cultivars that harbor health benefits. Accordingly, in this study, Tansawat et al. screened some Thai rice cultivars and discovered major volatile aroma-associated metabolites using gas chromatography–mass spectrometry (GC-MS). Moreover, their biosynthetic pathways were also studied. Their findings revealed 48 differentially accumulated metabolites among the 23 rice cultivars, among which the aromatic black rice cultivar harbored significantly higher levels of the two major groups of aroma-associated compounds, namely, aldehydes and alcohols.

In northwestern China, the wrinkled pepper (*Capsicum annuum* L.) harbors a unique flavor. In this study (Zhang, J. et al.), a total of 19 pepper cultivars, comprising 15 wrinkled and 4 smooth-skinned ones, were profiled for aroma-associated volatile metabolites at the mature stage. Their findings showed 199 volatile compounds in the screened cultivars. Notably, wrinkled pepper contained a significantly higher average volatile content compared to the smooth one. Among the detected metabolites, 29 volatile compounds mainly consisting of aldehydes, alcohols, and esters were found as the key aroma-associated ones in peppers, with 2-isobutyl-3-methoxypyrazine as the dominant one.

## Natural plant extracts as potential feed additives

Recently, to produce livestock food, natural extracts from various plants have been well investigated as promising feed additives, such as the usage of plant essential oils. In this study, Zhang, Y. et al. evaluated the effects of some extracts, including cinnamaldehyde, carvacrol, and thymol complex (CCT), on the growth rates of piglets inoculated with lipopolysaccharide. The findings showed that the usage of CCT as feed additives significantly declined the diarrhea rates of the piglets. Moreover, it also positively affected the intestinal absorption function in the piglets.

## Effect of plant-associated microbiota on fruit quality

The effect of the plant microbiota on fruit growth and health has been well documented. However, our knowledge regarding its potential impact on fruit quality is still limited. In this work, Escobar Rodríguez et al. used five different cultivars of tomato (*Solanum lycopersicum* L.) that were grown in two different conditions, namely, in soil and in hydroponics, to study the possible role of the cultivation approach in the fruit flavor. The findings revealed that the cultivar had a much greater impact on the aroma profile than the cultivation method. Moreover, a microbiota-dependent accumulation of flavor-related metabolites was observed in the tomato fruits.

In summary, the aim of this Research Topic is to gain a better understanding of flavor-associated metabolites in PBFs and to bridge the gap between plant scientists and food industrialists so that they may develop superior PBFs.

## Author contributions

GK wrote the first draft. TT and SS revised the manuscript. All authors have read and approved the manuscript.
